# Comparative QTL analysis of early short-time drought tolerance in Polish fodder and malting spring barleys

**DOI:** 10.1007/s00122-013-2190-x

**Published:** 2013-09-22

**Authors:** Magdalena Wójcik-Jagła, Marcin Rapacz, Mirosław Tyrka, Janusz Kościelniak, Katarzyna Crissy, Katarzyna Żmuda

**Affiliations:** 1 Department of Plant Physiology, University of Agriculture in Krakow, Podłużna 3, 30-239 Kraków, Poland; 2 Department of Biochemistry and Biotechnology, Rzeszow University of Technology, Albigowa 472, 37-122 Albigowa, Poland

## Abstract

*****Key message***:**

**An effective approach for the further evolution of QTL markers, may be to create mapping populations for locally adapted gene pools, and to phenotype the studied trait under local conditions.**

**Abstract:**

Mapping populations of Polish fodder and malting spring barleys (*Hordeum vulgare* L.) were used to analyze traits describing short-time drought response at the seedlings stage. High-throughput genotyping (Diversity Array Technology (DArT) markers) and phenotyping techniques were used. The results showed high genetic diversity of the studied populations which allowed the creation of high-density linkage maps. There was also high diversity in the physiological responses of the barleys. Quantitative trait locus (QTL) analysis revealed 18 QTLs for nine physiological traits on all chromosomes except 1H in malting barley and 15 QTLs for five physiological traits on chromosomes 2H, 4H, 5H and 6H in fodder barley. Chromosomes 4H and 5H contained QTLs which explained most of the observed phenotypic variations in both populations. There was a major QTL for net photosynthetic rate in the malting barley located on chromosome 5H and two major QTLs for overall photochemical performance (PI) located on 5H and 7H. One major QTL related to photochemical quenching of chlorophyll fluorescence was located on chromosome 4H in fodder barley. Three QTL regions were common to both mapping populations but the corresponding regions explained different drought-induced traits. One region was for QTLs related to PSII photosynthetic activity stress index in malting barley, and the corresponding region in fodder barley was related to the water content stress index. These results are in accordance with previous studies which showed that different traits were responsible for drought tolerance variations in fodder and malting barleys.

**Electronic supplementary material:**

The online version of this article (doi:10.1007/s00122-013-2190-x) contains supplementary material, which is available to authorized users.

## Introduction

Drought tolerance is a very important yet problematic trait for plant breeders. Difficulties arise from its quantitative nature. Drought tolerance undergoes a very complex genetic control involving many genes with small effects which are greatly affected by the environment (Mir et al. 2012). Because of this, one of the most suitable methods for identifying genes that are involved in drought tolerance is the use of molecular markers for quantitative trait loci (QTLs). The QTLs can then be used to improve the drought tolerance of the particular plant. To be able to determine QTLs for a desired trait, a genetic linkage map is required. Genetic linkage maps are constructed in a four stage process: create a mapping population, identify polymorphisms, genotype the mapping population and the parents with chosen markers, and linkage analysis of the markers (Collard et al. 2005). The most commonly used mapping populations consist of 50–250 individuals that originate from a cross between genetically distant parents that differ for the analyzed trait (Mohan et al. 1997). Several types of mapping populations that can be used to create a genetic linkage map: a population consisting of F_2_ plants, a backcross mapping population (from a backcross between an F_1_ plant and one of the parents), a recombinant inbred mapping population (obtained by self-pollination of single F_2_ individuals for at least six generations) or a double haploid mapping population (consisting of double haploids generated from pollen or embryos) (Collard et al. 2005). In terms of simplicity and time needed to create a particular mapping population, F_2_ mapping populations often seem to be the best choice. It is crucial for polymorphism identification to choose appropriate markers that will allow the production of a high-density map with the least possible effort. The genetic linkage map needs to be combined with a thorough analysis of the phenotype for the trait of interest in the mapping population (known as phenotyping).

The results of previous QTL mapping studies of drought tolerance-associated traits in barley (*Hordeum distichon* L.) illustrate many problems in finding common regions responsible for drought adaptation (Teulat et al. 1998, 2001, 2002, 2003; Baum et al. 2003; Diab et al. 2004; Comadran et al. 2008; Guo et al. 2008; von Korff et al. 2008; Chen et al. 2010). Most of the problems resulted from either different genotypes being studied under different environmental and controlled drought conditions, or various drought tolerance indicators being used in phenotyping. Water deficit affects metabolism of the whole plant and, as a result many different physiological characteristics have been used as a measure of drought tolerance. The measures include, yield and growth analysis (Mathews et al. 2008; von Korff et al. 2008), CO_2_ assimilation rate (Lawlor and Cornic 2002), PSII (photosystem II) photochemical activity (Oucarroum et al. 2007), leaf water conservation (Chen et al. 2004), plasma membrane integrity (Babu et al. 2004), osmotic adjustment or relative water content (Lilley et al. 1996; Teulat et al. 1998; Serraj and Sinclair 2002), carbon isotope discrimination (Teulat et al. 2002), and resistance to paraquat (Altinkut et al. 2003). The major challenge in phenotyping for drought tolerance is to choose a set of parameters, which can identify genotypes that are better adapted to drought events occurring in the local environment, and then to create a high-throughput phenotyping system that can describe this trait in the best way possible (Tuberosa 2010; Mir et al. 2012). Absolute values of drought tolerance parameters are needed to describe the drought tolerance level of breeding materials; whereas, relative values are sufficient to compare between materials, also in drought tolerance QTL studies (Teulat et al. 1998, 2001). Relative changes of physiological parameters or traits (stressed/control) are widely used for comparing of tolerance/resistance to various stress factors between plants. For instance, relative changes of fluorescence parameters were used to arrange 30 soybean genotypes according to their chilling tolerance (Strauss et al. 2006). Dolstra et al. (1994) used similar method for evaluation of genetic variation of 67 inbred maize lines for resistance to photoinhibition of photosynthesis. Therefore in this study, we used a relative measurement system to compare a number of known breeding lines in a gene pool already preselected for yield. The present study focused on the tolerance of spring barley seedlings to short-time drought, because spring water deficit events present major problem for barley production in Poland (Budzyński and Szempliński 2003). It was found recently that different physiological characteristics are responsible for variations in the early short-time drought tolerance observed in Polish malting and fodder barleys (Rapacz et al. 2010). Parameters connected with drought-induced changes in cell membrane integrity, chlorophyll fluorescence, and carbon assimilation rates were highly differentiated between malting barley genotypes; whereas plant water status and transpiration rates differed in the drought response of fodder barleys (Rapacz et al. 2010). In both groups of breeding material a large variation in drought response was also observed, which indicates that it may be possible to select and combine different traits observed in Polish breeding barleys to improve their drought tolerance.

In the present study, we used QTL mapping to search for the genetic background of the variation in drought response observed by Rapacz et al. (2010), both inside and between malting and fodder barleys. To address these goals, the progeny of malting and fodder barleys, which were selected as contrasting in terms of drought tolerance by Rapacz et al. (2010), were used to create two mapping populations. We developed a high-throughput phenotyping system to measure parameters correlated with cell membrane stability, gas exchange, chlorophyll fluorescence, plant water status, and carboxylation intensity. For genotyping we used one of the most effective high-throughput genotyping platforms that are currently available, Diversity Array Technology (DArT) (Jaccoud et al. 2001).

## Materials and methods

### Plant material

Different patterns of physiological and molecular response to drought in malting and fodder barleys bred in Poland were reported in a previous study (Rapacz et al. 2010). In both groups separate physiological parameters were selected as the most differentiating between the drought-tolerant and drought-susceptible barleys. Thus, to study the genetic background of the different drought tolerances, we created two segregating populations. Parents with contrasting drought tolerance were chosen on the basis of previous study (Rapacz et al. 2010). Two pairs of crossings were: STH836 (susceptible, malting, STH3600 × Blask) × STH754 (tolerant, malting, Extract × STH3901) and MOB12055 (susceptible, fodder, MOB2310/91 × WW7931) × STH369 (cv. Suweren, tolerant, fodder, Stratus × Annabell). Seeds were obtained from two Polish breeding companies, Strzelce Plant Breeding (STH) and Danko Plant Breeding, Modzurow (MOB) branch. The high drought tolerance of the Suweren cultivar was confirmed by both company breeding data and farmers’ opinion (Strzelce Plant Breeding 2012). The progeny of about 20 crossings made between single plants in each combination were used to generate of the F_2_ plants that we used for genotyping. The crossings, as well as the growth of the F_1_ plants, were performed in an air-conditioned greenhouse. Single plants were grown in pots (ø16 × 20 cm) filled with a mixture of universal garden soil substrate (Ekoziem, Jurkow, Poland) and sand (2:1, v:v). The plants were watered as required and fertilized up to the end of flowering with Florovit multipurpose fertilizer (Inco, Góra Kalwaria, Poland) according to the manufacturer’s instructions. The temperature was 25 °C/17 °C (day/night), the photoperiod was natural (parents, sown in early April) or 14/10 h (day/night), and the irradiance was natural (parents) or 400 μmol m^−2^ s^−1^ (HPS lamps, SON-T + AGRO, Philips, Brussels, Belgium). Single heads of F_1_ plants were bagged to secure self-pollination. In the case of the fodder barley population, over 200 of the seeds were obtained from a single F_1_ plant; thus this progeny was chosen for further study. In the case of the malting barley population, the number of seeds from a single plant was insufficient; therefore, the progenies of two F_1_ plants were used. In each of the two populations 183 F_2_ plants were genotyped. F_2_ seeds were sown in an air-conditioned greenhouse in the middle of April, the temperature was 25 °C/17 °C (day/night), with natural photoperiod and light intensity. The pots and plant care were the same as we used during F_0_ and F_1_ growth. After collecting leaves for DNA isolation the pots were transferred to open-air conditions to generate F_3_ plants for phenotyping. Heads were bagged to ensure self-pollination.

### Genotyping

DNA was isolated from the parental plants and plants of the F_2_ populations with DNeasy Plant Mini Kit (Qiagen, Hilden, Germany) according to the manufacturer’s instructions. The populations were screened with Diversity Array Technology (DArT) markers type Barley PstI(BstNI) version 1.7. The high resolution array comprises 1,500 markers, which are polymorphic in a wide range of barley cultivars from all over the world, identified by surveying 10,000 loci from a PstI(BstNI) genomic representation of cultivated barley accessions (Wenzl et al. 2004) and about 1,000 markers identified by surveying 10,000 loci from a PstI(BstNI) genomic representation of wild barley accessions. The positional information for some of polymorphic DArT markers was based on the barley integrated map (Wenzl et al. 2006). The maps were additionally saturated by 31 simple sequence repeats (SSR) and 32 sequence tagged sites (STS) markers obtained by DArT markers sequences conversion (Fiust 2011).

### Genetic linkage map construction

The genotypic data for DArT markers were used to construct genetic linkage maps with JoinMap 4.0 (Van Ooijen 2006). Prior to map construction, structures of both mapping populations were checked with PAST software (Hammer et al. 2001), and segregation data were adjusted to remove sub-population bias. Marker linkage groups were selected at logarithm of odds (LOD) scores >3.0. The initial cluster groups that we obtained were subsequently merged at lower LODs on the bases of colinearity with the reference integrated map (Wenzl et al. 2006). Then, markers within the two groups were ordered using the maximum likelihood algorithm. Genetic distances between loci were calculated based on the Kosambi mapping function. The proposed sequence of markers was checked graphically and corrected by the replacement of single data of double crossing over with missing data. In the STH 754 × STH 836 population, the average maximum number of recombinations per individual was 9.9 and ranged from 7.2 for the 1H chromosome to 13.2 for the 5H. Similarly, in the MOB12055 × STH369 population the mean maximum number of recombinations per individual was 9.3. Finally, the assignment of linkage groups on chromosomes was checked against the previously published barley consensus maps (Aghnoum et al. 2010; http://www.wheat.pw.usda.gov). Markers located at the same map positions were considered to be redundant and were shortlisted for clarity of the results. Further, bins of markers were identified with IciMapping (Wang et al. 2012a, b).

### Phenotyping

#### Plant growth conditions and drought treatment

Drought response of the segregated populations was analyzed in F_3_ progeny obtained by self-pollination of the F_2_ plants used for genotyping. Thirty seeds of each F_3_ line were sown in six pots (5 dm^3^) with a mixture of clay, peat, and sand (3/2/1, v/v/v). Plant growth occurred in growth chambers with a fully controlled environment. During germination (four days in darkness) a constant temperature of 25 °C was maintained, after emergence during the next eight days the temperature was 25 °C/17 °C (day/night), with photoperiod 14/10 h (day/night), irradiance of 400 μmol m^−2^s^−1^ (HPS lamps, SON-T + AGRO, Philips), and 50 % air humidity. During their growth the plants were watered and fertilized once a week with Florovit (R) multipurpose fertilizer (Inco) according to the manufacturer’s instructions. Up to the four-leaf stage (18 days after emerging), soil water content was kept at 70 % maximum water capacity by every day adding of an appropriate amount of water. After this the watering was stopped and the maximum water capacity decreased gradually, reaching 32 % after 7 days, which was measured with HydroSense Soil Water Content Measurment System (Campbell Scientific, Thuringowa Central, Australia). Under these conditions, leaves of all of the genotypes showed symptoms of turgor loss. Lines were sown sequentially on a daily basis: 6–7 lines with three pots per line (18–21 pots/day equivalent to 270–315 plants/day). The remaining three pots from each line were a part of the second independent experimental series. The results from both experimental series were averaged and the number of replications indicated in this paper represents the sum.

#### Measurements of physiological characteristics

All parameters were measured after the last watering (w) and at the end of the drought treatment (d). Stress indexes (SI) for each of the physiological parameters measured, were calculated as: SI (%) = (d/w) × 100 %, based on the stress indexes developed by Bouslama and Shapaugh (1984).

Plasma membrane integrity was determined by means of an electrolyte leakage (EL) test as described in detail by Rapacz et al. (2010). Measurements were performed on the first (the oldest) leaves in 12 replications (two from each pot in both experimental series).

Water relations in leaves were characterized by means of water content (WC = (FW−DW)/DW*100 %); FW, fresh weight; DW, dry weight). The measurements were done in 12 replications (two from each pot in both experimental series). Water content was measured in both populations, although no correlation of this parameter with other stress indexes, and no statistically significant effect of genotype on this parameter were observed previously in malting barleys (Rapacz et al. 2010). On the other hand, in that study the difference in the water content stress index was statistically significant between parents (STH754 and STH836). Measurements were performed on the second leaves in 12 replications (two from each of the three pots in both experimental series).

The net photosynthetic rate (*A*) was measured in the middle part of the third leaf using an infrared gas analyzer (Ciras-1, PP Systems, Hitchin, UK) and Parkinson leaf chamber (PLC6), as described elsewhere (Rapacz et al. 2010). The controlled measuring conditions were: CO_2_ concentration of 400 μmol (CO_2_) mol^−1^ (air), 30 % relative humidity, irradiance of 500 μmol (quanta) m^−2^ s^−1^ and the leaf temperature of 25 °C. The measurements were performed in 10–12 replicates (five-six for each experimental series). To estimate the quantum yield of CO_2_ (ΦCO_2_), A was divided by the absorbed light intensity (PAR × 0.84). Photochemical efficiency was estimated by means of chlorophyll *a* (Chl) fluorescence measurements. Measurements were taken in the middle part of the third leaf using either a modulated fluorescence system FMS2 or a fast chlorophyll fluorescence induction kinetics fluorymeter Handy PEA (Hansatech, Kings Lynn, UK), as previously described in detail (Rapacz et al. 2010). Fluorescence induction kinetics was measured only in the malting barley population because neither statistically significant differences between parents nor a correlation with other parameters was observed in the group of fodder barleys, as discussed previously (Rapacz et al. 2010). After light adaptation of the leaf (about 5 min at 500 μmol (quanta) m^−2^ s^−1^ when the fluorescence signal (F_s_) became constant), the FMS2 system was used to calculate the following parameters: (1) the PSII antenna trapping efficiency (*F*′_v_/*F*′_m_,) where *F*′_v_ = *F*′_0_–*F*′_m_ (*F*′_0_ is the chlorophyll fluorescence yield when all of the PSII reaction centers and electron acceptor molecules are fully oxidized in a light adapted leaf, and *F*′_m_ is the maximum fluorescence yield in a light adapted leaf); (2) the photochemical light energy quenching coefficient (*q*_P_) as *q*_P_ = (*F*′_m_–*F*_s_)/(*F*′_m_–*F*′_0_) according to Schreiber et al. (1986); and (3) the quantum yield of electron transport at PSII as ΦPSII = (*F*′_m_ *–F*_s_)/*F*′_m_ (Genty et al. 1989). The measurements were performed in 12 replicates (six in each experimental series). The induction of a chlorophyll fluorescence signal was measured after 30 min of leaf dark adaptation in clips (Hansatech). The following parameters were calculated based on the theory of energy flow in PSII and using the JIP-test (Strasser and Tsimilli–Michael 2001). The energy absorbed in PSII antennas (ABS/CS), trapped in PSII reaction centers (TRo/CS), used for electron transport (ETo/CS) and dissipated from PSII (DIo/CS), as well as the maximum number of active reaction centers (RC/CSm), was calculated per excited leaf cross section (CS) together with the overall performance index of PSII photochemistry (PI). The measurements were performed in 22–25 replicates (11–13 for each experimental series).

### QTL analysis

The distribution of the physiological characteristics data were checked and, in most of the cases, the normal distribution hypothesis (Shapiro–Wilk test, *p* = 0.00001) was not rejected. For  %EL and ΦPSII/ΦCO_2_ before the analysis, data were further normalized by log10 (*x* + 1) (Shen et al. 2006). Composite interval mapping (CIM) analysis was performed using the QTL Cartographer 2.5 software (Wang et al. 2012a, b). After performing a 1000 permutation test, a LOD threshold of 2.5 was set to declare a QTL as significant. A walk speed of 1.0 cM was chosen for all QTL detections. QTL effects were estimated as the proportion of phenotypic variance (*R* ^2^) explained by the QTL. QTLs were considered as minor or major, by defining a major QTL as a QTL that explained more than 15 % of the phenotypic variance in a primary genetic analysis (Salvi and Tuberosa 2005).

## Results

### Phenotyping

Drought stress indexes for the physiological parameters studied were highly differentiated both between parents and in the segregating populations (Table 1). The biggest diversity was observed in EL, A and ΦPSII/ΦCO_2_ in both of the populations. The values of most of the measured parameters were not distributed evenly in the mapping populations. In both of the populations ΦPSII/ΦCO_2_ was a parameter which distribution was most skewed to the right (in the direction of high values) which means that it could be useful for discarding of susceptible to drought genotypes in the process of selection (electronic supplementary materials, Fig. S11–30). In malting barley, the drought-tolerant parent (STH754) was less affected by drought than the susceptible parent in all the studied parameters, with the exception of water content where this difference was not statistically significant (Table 1). In fodder barley, the values of the parameters studied were significantly less affected by drought in four out of seven cases.

**Table 1:** Values of SI (%) calculated for physiological parameters characterizing drought response of the studied mapping populations (F_3_ lines) of malting and fodder barleys together with the values observed in parents

Physiological parameter	Malting barley					Fodder barley				
	Tolerant parent	Susceptible parent	F_3_ lines			Tolerant parent	Susceptible parent	F_3_ lines		
	STH754	STH836	Min.	Max.	Mean	Suweren	MOB12055	Min.	Max.	Mean
WC	101.0	99.4	91.5	100.9	96.6	99.6*	96.8	93.9	101.1	97.4
EL	102.0*	387.1	92.1	1171.2	446.2	130.4*	280.8	142.4	1428.1	561.2
*A*	13.7*	8.8	1.2	20.8	9.3	13.4	15.0	0.3	56.7	9.0
*F*′v/*F*′m	84.8*	76.5	71.9	100.1	87.5	84.3	83.1	73.3	94.0	82.7
*q*_P_	79.8*	66.9	50.8	96.8	80.0	83.4*	80.3	59.3	111.4	83.2
ΦPSII	84.7*	76.7	71.9	100.2	87.5	67.8	69.2	45.2	96.6	69.2
ΦPSII/ΦCO_2_	660.2*	742.0	469.2	7423.2	1664.4	500.1*	938.6	155.7	35858.3	2637.0
ABS/CS	72.5*	71.8	56.6	95.2	74.8	–	–	–	–	–
TRo/CS	67.4*	65.5	53.8	100.0	71.5	–	–	–	–	–
ETo/CS	54.5*	49.4	39.4	70.1	54.3	–	–	–	–	–
DIo/CS	89.9*	92.6	63.9	98.6	85.1	–	–	–	–	–
RC/CSm	70.9*	64.1	55.4	104.2	79.1	–	–	–	–	–
PI	33.4*	25.9	17.6	72.1	38.1	–	–	–	–	–

SI values represent the  % of the parameter value in drought relative to the value measured before drought. Please consider that in the case of EL, ΦPSII/ΦCO_2_ and DIo/CS higher values of the parameter means higher drought susceptibility, whereas in the remaining cases higher tolerance. The distribution of SI values in segregating population was always normal according to Shapiro–Wilk test (*P* = 0.05) with the exception of SI for ΦPSII/ΦCO_2_. SI values for tolerant parents indicated with the *asterisk* are significantly different than for susceptible one according to Mann–Whitney’s *U* test (the mean for genotype before drought was used for calculation of SI independently for each replication during drought) for *P* = 0.05

### Genotyping

**Fig. 1:**
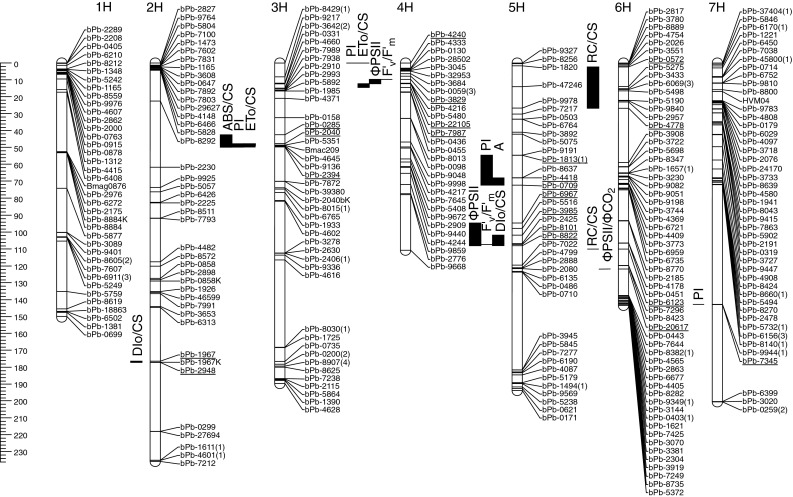
Linkage map for 331 DArT, 4 STS and 3 SSR markers reduced to 295 unique loci based on 182 F_2_ plants derived from the cross between malting barleys: STH754 × STH836 and position of quantitative trait loci (*QTL*) for stress indexes (*SI*) of different physiological characteristics. Genetic distances are shown in centiMorgans (*cM*) to the *left* of the *vertical axis*.* Numbers in brackets* refer to redundant markers

**Fig. 2:**
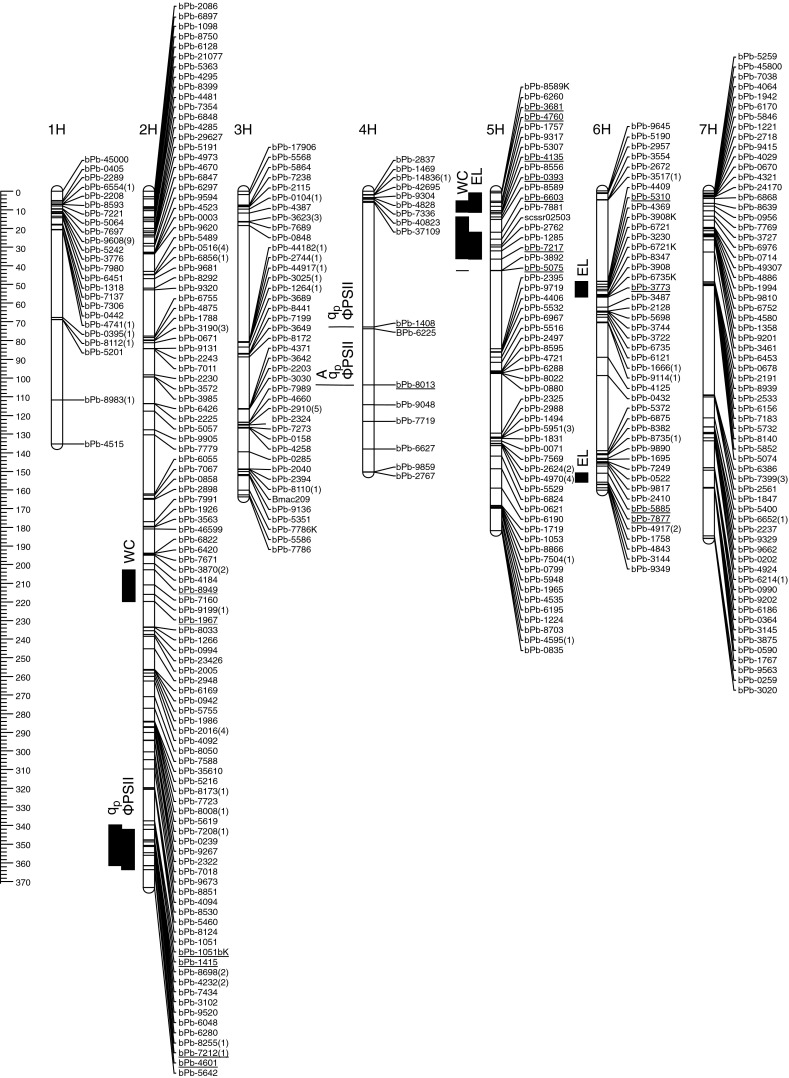
Linkage map for 423 DArT, 6 STS and 2 SSR markers representing 335 loci based on 182 F_2_ plants derived from the cross between fodder barleys: Suweren × MOB12055 and position of quantitative trait loci (*QTL*) for stress indexes (*SI*) of different physiological characteristics. Genetic distances are shown in centiMorgans (*cM*) to the *left* of the *vertical axis*.* Numbers in brackets* refer to redundant markers

DArT markers for 183 individuals of the STH 754 × STH 836 population and parents were scored for segregation on a panel of 2500 PstI(BstNI) genomic representations of barley. Genotyping of the STH754 × STH836 malting barley population revealed 373 polymorphic DArTs; 301 of them were polymorphic between parents, and two variant phases were tested for the remaining 72 markers. The distribution of genotypes across the principal coordinates suggested the presence of two subpopulations corresponding to progenies obtained from two independent crosses. In the course of the mapping, 45 markers monomorphic within subpopulations were replaced with missing data, and 43 markers were deleted. Finally, 331 DArTs, four STS and three SSR markers that reduced to 295 unique loci were used to create a 1226 cM long genetic map for STH754 × STH836 malting barley (Fig. 1). Similarly, a1362 cM long genetic map was constructed for fodder barley Suweren × MOB12055 based on the segregation of 423 DArT, six STS and two SSR markers representing 330 loci (Fig. 2). First, we obtained segregation data for 452 DArT markers including 392 markers polymorphic on parental forms and 60 markers of unknown phase. For both populations, the order of the markers on the map was in perfect agreement with the reference data. Although the markers were not evenly distributed on the chromosomes, they covered the chromosomes with a density of 4.16 and 4.13 cM/marker for the malting and fodder barley, respectively, providing a good framework for the subsequent QTL mapping. The two mapping populations had 120 DArT markers in common. Clusters of markers were in agreement between the two maps, and only minor differences in marker order were found on chromosomes 6H and 7H.

### QTL analysis

The CIM analysis for the malting barley mapping population revealed 18 QTLs (15 minor and 3 major) for nine physiological traits among all of the chromosomes except 1H (Table 2, Fig. 1). For the fodder barley population, 15 QTLs (14 minor and 1 major) for five physiological traits were found on chromosomes 2H, 4H, 5H and 6H (Table 3, Fig. 2). In both populations the 4H and 5H chromosomes contained regions that explained most of the observed phenotypic variation in the parameters analyzed. No QTLs for the water content stress index were found in the malting barley population. In the fodder barley population, 4 QTLs for the water content stress index were detected on chromosomes 2H and 5H, explaining 37.35 % of the observed phenotypic variation with the largest contribution from markers bPb-1967 (2H) and bPb-5075 (5H) (Table 3). No QTLs for the drought-induced membrane damage (EL) or for the photochemical light energy quenching coefficient (*q*_P_) were detected in the malting barley mapping population, while four QTLs for EL and three for q_P_ change were found in the fodder barley population (Table 3). On the other hand, the fodder barley QTL map lacked the QTLs for the *F*′_v_/*F*′_m_ stress index, which were present on chromosomes 4H and 5H in the malting barley map (Table 2). QTLs were found in both populations for net photosynthetic rate (*A*) and quantum yield of electron transport at PSII (ΦPSII). One major QTL (*QA.sthb*-*5H*), which explained 22.19 % of the observed phenotypic variation in A in the malting barley mapping population was located on chromosome 5H in the vicinity of markers bPb-4418 and bPb-0709 (Table 2). In the fodder barley population also, one QTL (*QA.sthf*-*4H*, area of marker bPb-8013) that explained 7.25 % of the observed phenotypic variation in A was detected, but this QTL was located on chromosome 4H (Table 3). Two QTLs (*QPSII.sthb*-*4H* and *QPSII.sthb*-*5H*), which explained 17.79 % of phenotypic variation in drought-induced change in ΦPSII, were detected on chromosomes 4H and 5H in the malting barley mapping population. The biggest effects originated from markers bPb-8101 and bPb-8822 on chromosome 5H (Table 2). Three QTLs for this trait were found in the fodder barley population on chromosomes 2H and 4H, and together they explained 27.76 % of the observed phenotypic variation in ΦPSII. Among the markers present in these regions marker bPb-8013 located on chromosome 4H contributed the most to the observed effect (Table 3). In addition, QTLs *QPSII.sthm*-*4H* in the malting barley mapping population and *QPSII.sthf*-*4H.2* in the fodder barley mapping population were located in the corresponding positions of 60.0 cM and 69.7 cM, respectively on the consensus reference map (Tables 2 and 3; Figs. 1 and 2). QTLs for the chlorophyll fluorescence parameters that describe energy flows and energy transfer efficiencies in PSII (JIP-test parameters) were mapped only in the malting barley population. On chromosomes 3H, 4H, 5H and 6H these QTLs overlapped or co-located either with the QTLs for other JIP-test parameters or with QTLs for chlorophyll fluorescence parameters as measured by gas exchange. In addition, QTL *QPI.sthm*-*5H* overlapped with the net-assimilation rate QTL *QA.sthm*-*5H.* PI, which characterizes the overall performance index of light energy use in PSII seems to be controlled by two main genes, one on chromosome 5H (*QPI.sthm*-*5H*), which explained 46.92 % of the observed phenotypic variation (marker bPb-1813(1)), and the other on chromosome 7H (*QPI.sthm*-*5H*), which explained 52.01 % of the observed variation (marker bPb-7345). However, two additional QTLs with smaller effects were found on chromosomes 2H and 3H.

**Table 2:** QTLs for stress indexes (SIs) for different physiological traits studied in the mapping population of malting barley (STH754 × STH836); bold letters indicate major QTLs

Trait	Linkage group	Flanking markers	QTL^a^	LOD^b^	Add^c^	*R* ^2^ (%)^d^
Gas exchange						
* A*	5H	bPb-4418	***QA.sthm*** **-** ***5H***	**2.64**	**2.247**	**22.19**
		bPb-0709		**2.75**	**2.386**	**21.29**
Photochemical activity of PSII						
* F*′_v_/*F*′_m_	4H	bPb-3829	*QFvFm.sthm*-*4H*	2.51	−0.224	6.32
	5H	bPb-8101	*QFvFm.sthm*-*5H*	2.52	2.846	9.79
ΦPSII	4H	bPb-3829	*QPSII.sthm*-*4H*	2.56	−0.239	6.66
		bPb-22105		2.57	−0.239	6.22
	5H	bPb-6967	*QPSII.sthm*-*5H*	2.53	3.467	8.03
		bPb-3985		2.50	3.083	8.11
		bPb-8101		2.71	3.094	11.13
		bPb-8822		2.68	3.046	11.38
ΦPSII/ΦCO_2_	6H	bPb-20617	*QPSII*-*CO2.sthm*-*6H*	5.49	298.943	5.66
ABS/CS	3H	bPb-0285	*QABS*-*CS.sthm*-*3H*	2.87	−2.7187	8.23
		bPb-2040		3.16	−2.709	8.29
		Bmac209		3.18	−2.7247	8.24
		bPb-2394		3.06	−2.7005	8.19
ETo/CS	3H	bPb-2040	*QET.sthm*-*3H*	2.62	−2.626	7.93
		Bmac209		2.68	−2.6529	7.97
		bPb-2394		2.64	−2.640	8.00
	4H	bPb-4240	*QET.sthm*-*4H.1*	2.58	−1.870	6.05
	4H	bPb-22105	*QET.sthm*-*4H.2*	2.59	−168.085	5.80
		bPb-7987		2.63	−166.049	6.23
DIo/CS	2H	bPb-1967	*QDI.sthm*-*2H*	3.83	1.857	9.08
		bPb-2948		3.70	1.789	8.25
	5H	bPb-3985	*QDI.sthm*-*5H*	2.56	−0.184	8.03
		bPb-8101		3.07	−0.268	10.84
		bPb-8822		3.14	−0.288	11.33
RC/CSm	6H	bPb-0572	*QRC*-*CS.sthm*-*6H.1*	3.46	0.000	7.33
		bPb-4778		3.10	−2.727	7.13
	6H	bPb-6123	*QRC*-*CS.sthm*-*6H.2*	2.51	−3.063	5.09
PI	3H	bPb-2040	*QPI.sthm*-*3H*	2.78	−3.490	7.46
		Bmac209		2.85	−3.515	7.67
		bPb-2394		2.86	−3.498	7.85
	4H	bPb-4240	*QPI.sthm*-*4H*	2.60	−4.216	5.47
	5H	bPb-1813(1)	***QPI.sthm*** **-** ***5H***	**2.87**	**0.000**	**46.92**
		bPb-4418		**2.92**	**0.000**	**43.33**
		bPb-0709		**3.04**	**0.000**	**43.84**
	7H	bPb-7345	***QPI.sthm*** **-** ***7H***	**5.62**	**0.000**	**52.01**

^a^QTL were detected with a minimum LOD score of 2.5 in at least one environment

^b^Logarithm of odds (LOD) score

^c^Additive effect of allele

^d^Percentages of phenotypic variance explained by individual QTL

**Table 3:** QTLs for stress indexes (SIs) for different physiological traits studied in the mapping population of fodder barley (Suweren × MOB12044); bold letters indicate major QTLs

Trait	Linkage group	Flanking markers	QTL^a^	LOD^b^	Add^c^	*R* ^2^ (%)^d^
Water relations						
WC	2H	bPb-8949	*QWC.sthf*-*2H*	3.25	0.135	8.10
		bPb-7160		3.78	0.716	9.64
		bPb-9199(1)		4.59	0.748	10.08
		bPb-1967		4.16	0.764	10.16
	5H	bPb-4760	*QWC.sthf*-*5H.1*	2.61	7.346	5.92
		bPb-1757		2.79	7.338	6.23
		bPb-9317		3.14	2.320	6.36
		bPb-5307		2.84	7.347	6.16
		bPb-4135		2.59	7.342	5.46
	5H	bPb-0393	*QWC.sthf*-*5H.2*	2.64	0.036	5.31
		bPb-8589		2.89	0.097	6.03
		bPb-6603		4.22	0.254	9.04
		bPb-7881		4.44	0.273	9.47
		scssr02503		4.00	0.248	7.87
		bPb-2762		3.66	0.187	7.54
		bPb-1285		3.10	0.102	6.56
		bPb-7217		2.55	1.108	5.32
		bPb-5075	*QWC.sthf*-*5H.3*	3.25	0.330	11.36
Membrane integrity						
EL	5H	bPb-3681	*QEL.sthf*-*5H.1*	2.80	−122.887	5.59
		bPb-4760		3.01	0.000	7.38
		bPb-1757		3.17	0.000	7.83
		bPb-9317		3.31	0.000	7.16
		bPb-5307		2.97	0.000	6.97
		bPb-4135		2.77	0.000	6.14
	5H	bPb-6603	*QEL.sthf*-5H.2	4.19	−101.903	9.27
		bPb-7881		3.53	−90.423	8.08
		scssr02503		2.90	−69.031	6.07
		bPb-2762		3.13	−73.352	6.71
		bPb-1285		3.19	−82.241	6.94
		bPb-7217		3.07	0.000	6.99
	6H	bPb-5310	*QEL.sthf*-*6H.1*	2.47	−94.089	5.61
		bPb-4369		3.07	−110.376	7.28
		bPb-6721		3.10	−106.096	6.96
		bPb-3230		2.84	−103.295	6.39
		bPb-6721 K		2.94	−106.068	6.65
		bPb-8347		3.14	−104.703	7.08
		bPb-6735 K		3.15	−103.251	7.06
		bPb-3773		3.17	−103.655	7.21
	6H	bPb-5885	*QEL.sthf*-*6H.2*	2.52	5.001	6.27
		bPb-7877		2.68	4.561	6.17
Gas exchange						
A	4H	bPb-8013	*QA.sthf*-*4H*	2.93	−3.315	7.25
Photochemical activity of PSII						
q_P_	2H	bPb-1051bK	*Qqp.sthf*-*2H*	2.82	4.180	5.85
		bPb-1415		2.72	4.281	5.68
		bPb-4232(2)		2.79	4.775	5.80
		bPb-7434		3.05	4.996	6.43
		bPb-9520		3.46	5.437	7.13
		bPb-6048		3.48	5.493	7.20
		bPb-6280		3.49	5.487	7.23
		bPb-7212(1)		2.87	4.788	5.96
	4H	bPb-1408	*Qqp.sthf*-*4H.1*	4.00	6.480	8.66
	4H	bPb-8013	***Qqp.sthf*** **-** ***4H.2***	**5.81**	**−6.381**	**15.13**
ΦPSII	2H	bPb-1415	*QPSII.sthf*-*2H*	2.96	4.924	6.25
		bPb-4232(2)		2.80	5.104	5.93
		bPb-7434		2.99	5.267	6.39
		bPb-9520		3.32	5.660	7.02
		bPb-6048		3.40	5.738	7.27
		bPb-6280		3.41	5.733	7.31
		bPb-7212(1)		2.90	5.104	6.13
		bPb-4601		2.90	5.086	6.22
	4H	bPb-1408	*QPSII.sthf*-*4H.1*	3.08	6.184	6.60
	4H	bPb-8013	*QPSII.sthf*-*4H.2*	5.26	−6.90	13.85

^a^QTL were detected with a minimum LOD score of 2.5 in at least one environment

^b^Logarithm of odds (LOD) score

^c^Additive effect of allele

^d^Percentages of phenotypic variance explained by individual QTL

Further, two regions that correlated with drought tolerance were common for both mapping populations; however, they were located on chromosomes 2H and 6H that controlled different traits in each population. The vicinity of marker bPb-1967 on chromosome 2H was related to drought-induced water content change in the fodder barley mapping population, whereas in the malting barley population it was connected to changes in DIo/CS, a parameter that described PSII photochemical activity. The second region that was common for both populations was located on chromosome 6H and flanked by markers bPb-5190 and bPb-2957. This region was related to changes in RC/CS in malting barley and in ΦPSII/ΦCO_2_ in fodder barley (Figs. 1 and 2). Neither QTLNetwork (Yang et al. 2008) nor IciMapping (Wang et al. 2012a, b) revealed significant interactions (LODs ranged from 3.7 to 19.7) between QLTs that were identified using the CIM procedure of QTL Cartographer 2.5 (Wang et al. 2012a, b).

## Discussion

Over the last decade, barley has been the subject of extensive mapping studies with the DArT technology. A total of 2,032 DArT markers have been mapped to 646 unique positions (bins) in *Hordeum chilense* recombinant inbred line (RIL) population (Rodríguez-Suárez et al. 2012). The map based on segregations in progeny from a cross of the Igri and Franka cultivars representing separate gene pools of two- and six-rowed barley contained 527 DArTs (Sharma et al. 2011). The numbers of DArT bPb markers in double haploid and RIL populations that were used for consensus mapping varied from 257 to 530 (Wenzl et al. 2006). Alsop et al. (2011) reported similar numbers of mapped DArT loci in four populations of barley. The number of markers varied from 620 to 551 and number of bins was between 240 and 182, respectively. Based on these findings, we have mapped 88.7 and 93.6 % of the primary polymorphic DArT markers in the malting and fodder barley breeder populations, respectively, resulting in initial genetic maps built of 338 and 355 bin markers. However, a further reduction of markers with the highest number of missing data was still possible in these populations, resulting in 163 and 211 bins in the malting and fodder barley populations, respectively. In previous mapping studies of barley, 457 of 557 DArT markers (83 %) were used for genetic map construction (Grewal et al. 2008).

The high efficiency of mapping markers is of paramount importance in species that have a variable genomic constitution and possible chromosome rearrangements. In the genetic map of triticale, only 6 % of the predominant DArT markers remained unmapped (Tyrka et al. 2011). At present, DArT, as a system of choice for the generation of high-density maps, and new sequencing based technologies have evident advantages over hybridization-based DArT arrays (Poland et al. 2012). DArT markers are dominant, which in a hybridization-based system may lead to erroneous analysis of missing data, allele absence, and presence of ‘null’ alleles. Genotyping errors can be partially recognized as “singletons” during the mapping procedure and replaced by missing data. In comparison to new generation genotyping by sequencing systems in barley, DArTs have limited sequence information and 2000 bPb markers are publicly accessible. In association studies the biallelic DArT markers may result in more spurious associations than multiallelic markers.

Mapping of QTLs on segregating populations derived from breeding lines should result in the identification of loci, which are important for improvement of lines, but may suffer from incomplete homozygosity of parental lines and narrow genetic distances. These factors may also cause insufficient marker density in selected chromosomal regions. In spite of the large gaps that are present in the genetic maps of malting and fodder barley, conservation of the order of the loci with respect to the consensus map allows comparative localization of QTLs to be performed. However, insufficient saturation with markers may result in some important regions containing QTLs being missed. QTL mapping on an F_2_ population and later on recombinant inbreed lines resulted in the identification of additional QTLs in population of RILs (Myśków et al. 2012).

The genetic and phenotypic variations observed in the mapping populations that were created for the present study were enough to create high-density linkage and QTL maps. The newly constructed maps confirmed the accuracy of the selection criteria for early short-term drought tolerance in spring barleys previously proposed by Rapacz et al. (2010) and implemented in our study to choose parents for the populations. The average distance between loci was 4.16 cM for the malting and 4.03 cM for the fodder barley map. The average distance between the loci in the other populations that were mapped for drought tolerance QTLs was considerably larger; for example, 12.2 (Diab et al. 2004) and 14.5 cM (Teulat et al. 1998). The high density of the genetic maps that were created in this study showed that there was large genetic diversity and variation in the analyzed phenotypic traits, in spite of the fact that all the breeding strains studied were bred in Poland and originated from middle and northern European cultivars [six Polish, one German (Annabell), and one British (Extract)]. The obtained high density of the maps was also the result of our choice of marker system, namely, the large number of markers that we tested; about 2,500 (http://www.triticarte.com.au). No other marker system currently available can compete with this high number of markers. The degree of genetic diversity, as well as the diversity in drought-related traits that we observed for the mapping populations in this study, are similar to the degrees of diversity reported in other popularly used mapping populations; for example, double haploid population Proctor × Nudinka (Heun et al. 1991), double haploid population Steptoe × Morex (Kleinhofs et al. 1993), and the recombinant inbred population Tadmor × Er/Apm (Teulat et al. 1998, 2001).

The diversity of the phenotypic traits that we observed in the mapping populations of in the present study indicated that the sources of drought tolerance, which already existed in the Polish breeding materials of barley, were enough for the improvement of their tolerance to the spring droughts that frequently occur in Poland. Therefore, there is no need to search for external sources of drought tolerance in, for example, wild barley relatives or in local cultivars grown in a dry environment. Cattivelli et al. (2008), in their review of the improvement of drought tolerance in crops, stated that the biggest challenge for plant breeders is to introduce QTLs, which were obtained for a given mapping population into high-yielding elite genotypes. The results of our study suggest that an alternative approach based on the creation of mapping populations from high-yielding genotypes adapted to local conditions may be effective. Therefore, either advanced breeding lines or cultivars could be used. This approach is likely to accelerate breeding; first, because of the high correlation between drought tolerance, and yield (Comadran et al. 2008), and grain quality (Rapacz et al. 2010) and, second, because of the substantial influence of the environment. The high impact of local conditions on drought tolerance traits was shown for the cross between tolerant to drought Tadmor and Er/Apm (adapted only to specific dry conditions) mapping population mentioned above, tested in four different locations over four years (von Korff et al. 2008).

A precise comparison between different barley QTL maps is, of course, impossible because of the high specificity of the physiological and molecular determinants of drought tolerance which depend on the analyzed gene pool and environmental conditions. The gene pool of Mediterranean barley is perhaps the best described in terms of QTLs for drought tolerance. Diab et al. (2004) determined several QTLs for osmotic adaptation, relative water content, and leaf osmotic potential in the Tadmor × Er/Apm mapping population. The QTLs were located on all of the chromosomes, but mostly on 3H and 5H. In the same mapping population, Teulat et al. (1998) found QTLs which explained most of the observed variation in traits relating to plant water status on chromosome 6H. In the present study, we found QTLs for these traits were determined only in the fodder barley mapping population and on chromosomes 2H and 5H. The differences in QTL distribution and the percentage of explained phenotypic variations on various QTL maps of the different barley cultivars resulted mostly from genotype × environment interactions, which are known to greatly affect quantitative traits.

Genotype × environment interactions make it difficult to compare drought tolerance QTLs that have been determined under different environmental conditions and for different genotypes. However, we found some QTL regions that were common for the malting and fodder barley maps as well as for barley QTL maps that published previously. Common regions on the malting and fodder barley maps were located on chromosomes 2H, 4H and 6H. Of the QTLs that overlapped in both populations, the largest number was on chromosome 4H and most of these overlapping QTLs were for photochemical efficiency of PSII. In other studies, QTLs correlated with drought tolerance in barley were also found on chromosomes 2H, 4H and 6H; for example, QTLs for chlorophyll fluorescence induction kinetics, days to heading, plant height (von Korff et al. 2008), grain yield (Comadran et al. 2008, von Korff et al. 2008) and osmotic adjustment (Teulat et al. 1998, 2001). What is more, in the fodder barley mapping population, we found that co-localizing QTLs for leaf water content and electrolyte leakage were located together on the chromosome 5H in a region that harbors the *HVABI5* gene that is involved in plant response to abscisic acid (Tondelli et al. 2006). In the malting barley mapping population also, we found overlapping QTLs for chlorophyll fluorescence parameters on this chromosome (5H). Many studies for different traits have reported the location of different genes and QTLs in the same chromosomal regions (2H, 4H, 5H, and 6H) suggesting that these regions are correlated with barley’s response to drought irrespective of line/cultivar, origin, or environmental conditions. Therefore, the genetic background of drought tolerance is, in a broad sense, probably the same for barley as a species, while the role of particular genetic determinants in the expression of drought tolerance observed between genotypes depends on the gene pool and local environmental conditions. In the present study, these trends were also observed. Although many different QTLs were found for each mapping population, the presence of common regions in the chromosomes of the studied barleys confirmed that there was a similar background of spring drought tolerance in these populations. In the fodder barley population, QTLs that explained most of the observed phenotypic variation for leaf water content were identified, whereas, in the malting barley mapping population, no QTL for drought-induced changes in plant water status was found. What is more, among the three chromosomal regions correlated with drought tolerance that were common to both mapping populations, two of the regions were for different traits in the two populations. One region was for QTLs related to PSII photosynthetic activity stress index in malting barley, and the corresponding region in fodder barley was related to the water content stress index. These results confirm the observations made by Rapacz et al. (2010) that the differences in a drought-induced decrease in leaf water content represented one of the main elements of the differential drought response observed among fodder barley genotypes, whereas the differences were not significant among malting barley genotypes. On the contrary, stress indexes connected with drought-induced changes in energy fluxes and their efficiencies inside PSII (JIP-test parameters) were different only between malting barley genotypes. Therefore, it can be assumed that the same genetic determinants with putative regulatory functions may cause different changes in phenotype when acting in a different genetic background.

In conclusion, the wide diversity in drought tolerance among advanced barley breeding lines indicates the possibility of selection for early short-term drought tolerance within Polish spring barleys. For QTL analysis, an effective approach for the further evolution of functioning marker systems to improve the drought tolerance of barley, may be, first, to create separate mapping populations for locally adapted gene pools for different breeding directions (for example, for malting and fodder barleys), and, then, to phenotype the well-recognized physiological characteristics that are responsible for variations in drought tolerance among the studied genotypes and under local conditions. This approach should to be both a fast and easy one when using high-throughput genotyping and phenotyping methods.

## Electronic supplementary material

Below is the link to the electronic supplementary material.

Supplementary material 1 (DOCX 804 kb)
