# Supplementary material for: Comparative QTL analysis of early short-time drought tolerance in Polish fodder and malting spring barleys
Source: Theor Appl Genet. 2013 Sep 22;126(12):3021–34. doi: 10.1007/s00122-013-2190-x (PMC3838596; doi:10.1007/s00122-013-2190-x)
Supplement: Supplementary file 1 — Supplementary material 1 (DOCX 804 kb) [file 122_2013_2190_MOESM1_ESM.docx]

Fig S1. Distribution of LOD scores and additive variance (lower diagram) for trait DIo/CS along chromosome 2H of malting barley.


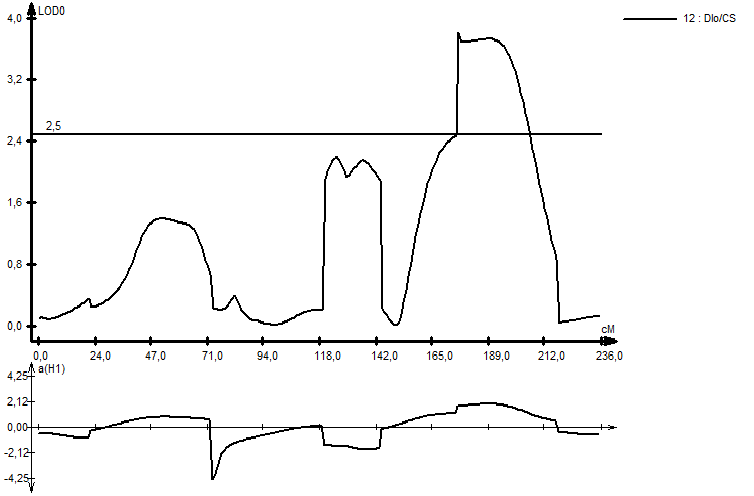


Fig S2. Distribution of LOD scores and additive variance (lower diagram) for traits ABS/CS, TRo/CS, ETo/CS, and PI along chromosome 3H of malting barley.


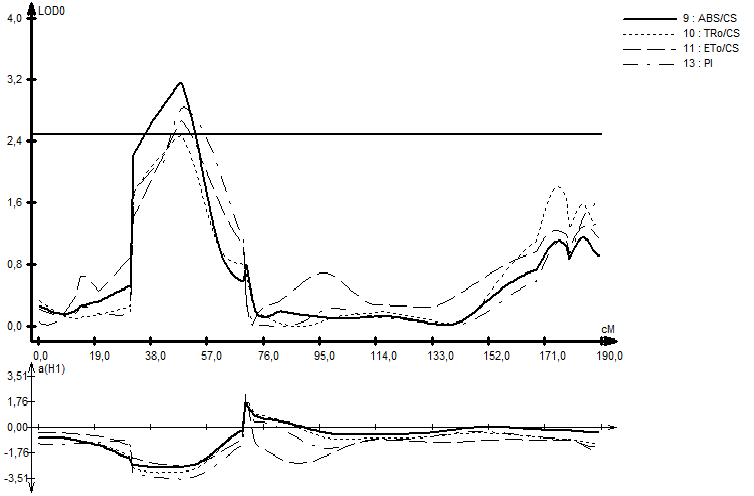


Fig S3. Distribution of LOD scores and additive variance (lower diagram) for traits F’_v_/F’_m_, ΦPSII, TRo/CS, ETo/CS and PI along chromosome 4H of malting barley.


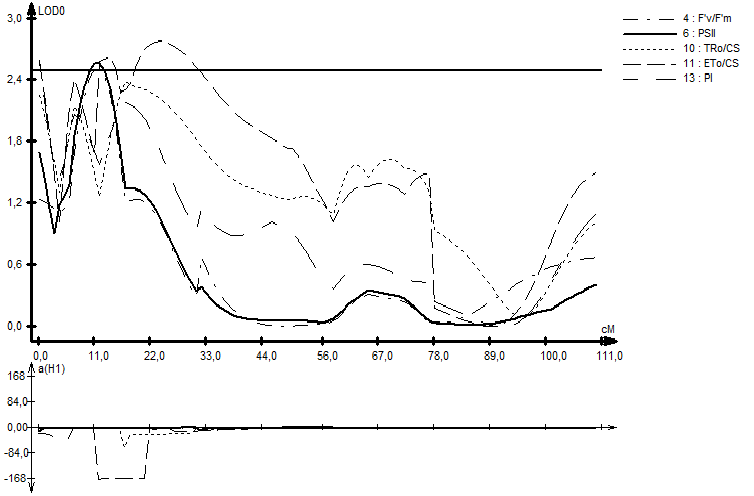


Fig S4. Distribution of LOD scores and additive variance (lower diagram) for traits A, F’_v_/F’_m_, ΦPSII, DIo/CS and PI along chromosome 5H of malting barley.


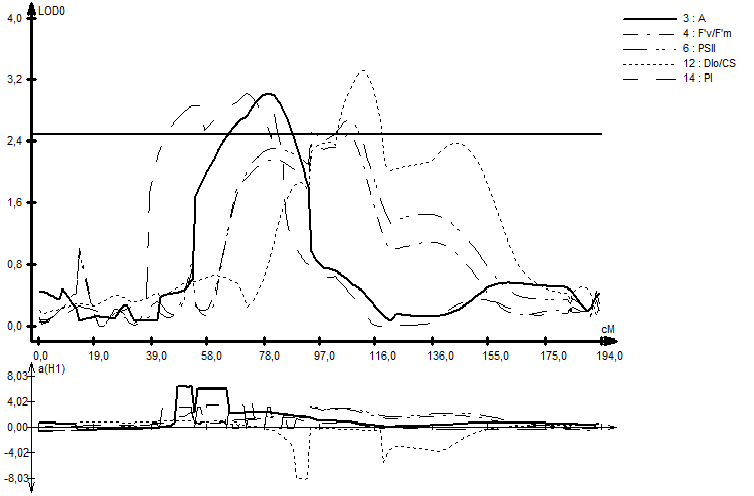


Fig S5. Distribution of LOD scores and additive variance (lower diagram) for traits ΦPSII/ΦCO_2_ and RCo/CS along chromosome 6H of malting barley.


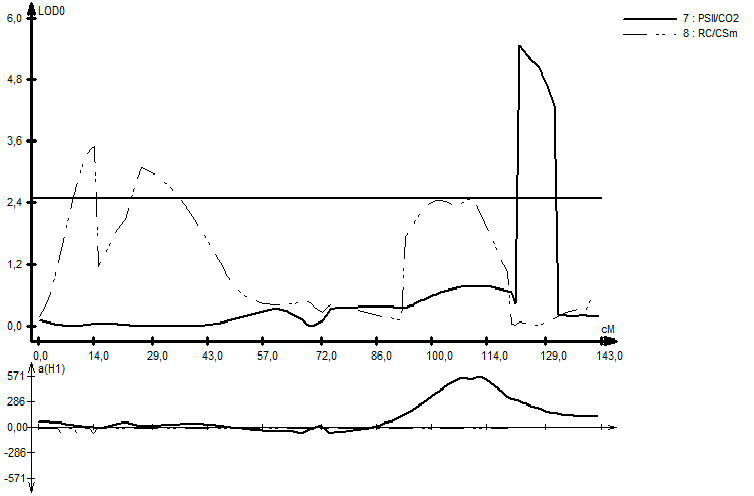


Fig S5. Distribution of LOD scores and additive variance (lower diagram) for trait PI along chromosome 7H of malting barley.


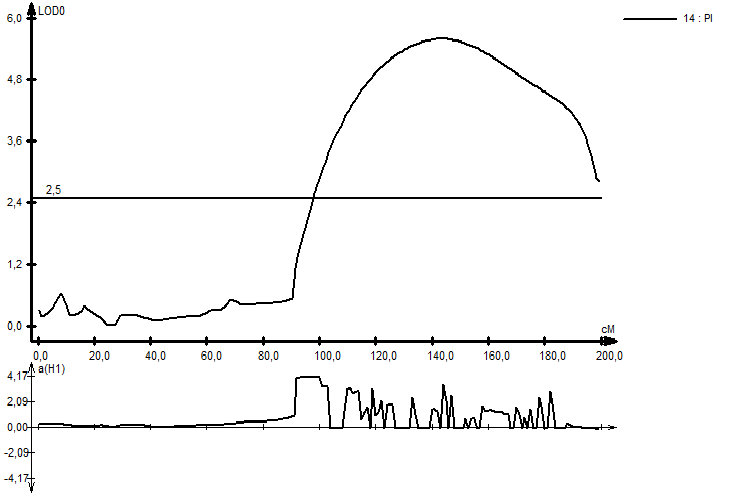


Fig S7. Distribution of LOD scores and additive variance (lower diagram) for traits WC and q_P_ along chromosome 2H of fodder barley.


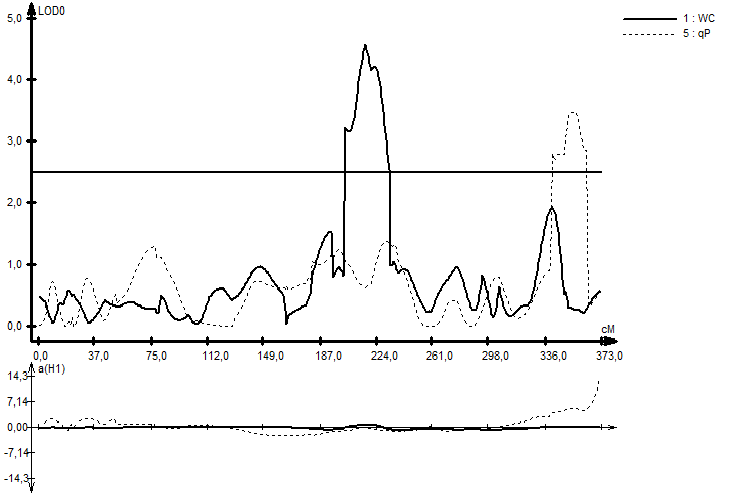


Fig S8. Distribution of LOD scores and additive variance (lower diagram) for traits A, q_P_ and ΦPSII along chromosome 4H of fodder barley.


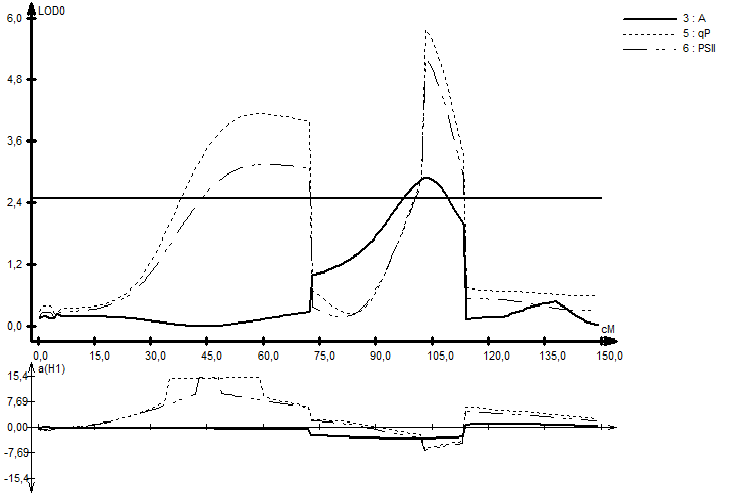


Fig S9. Distribution of LOD scores and additive variance (lower diagram) for traits WC and EL along chromosome 5H of fodder barley.


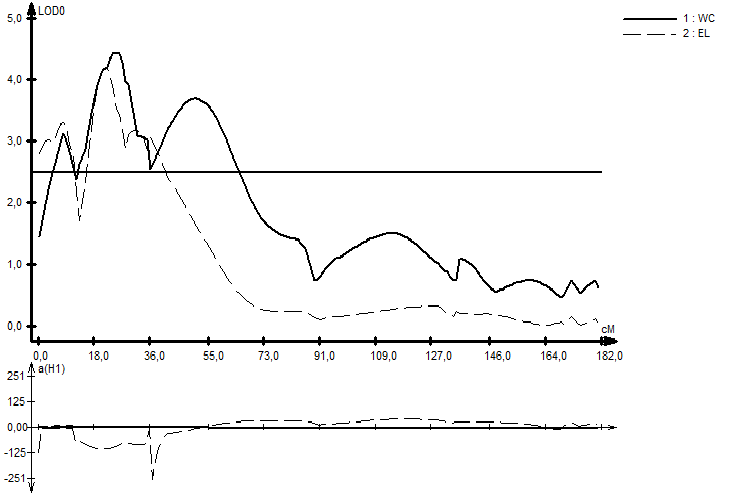


Fig S10. Distribution of LOD scores and additive variance (lower diagram) for trait EL along chromosome 6H of fodder barley.


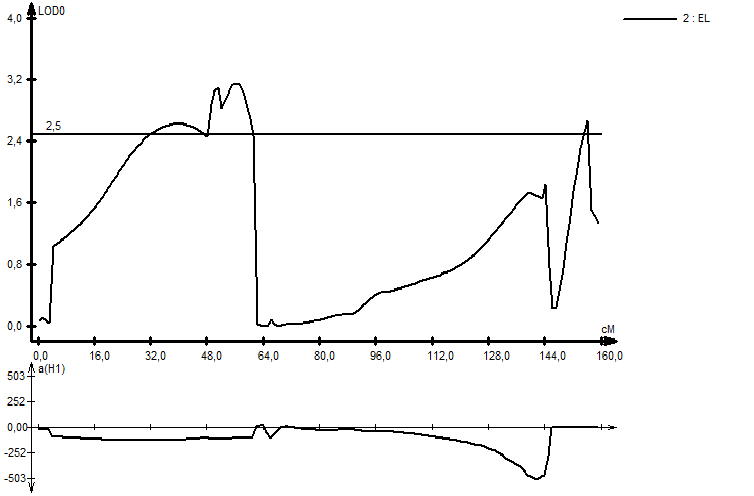


Fig S11. Histogram of stress index values for electrolyte leakage (ELSI) measured during phenotyping of F_3_ progeny in malt-type barley mapping population.

*

**

STH 836 *

STH 754 **


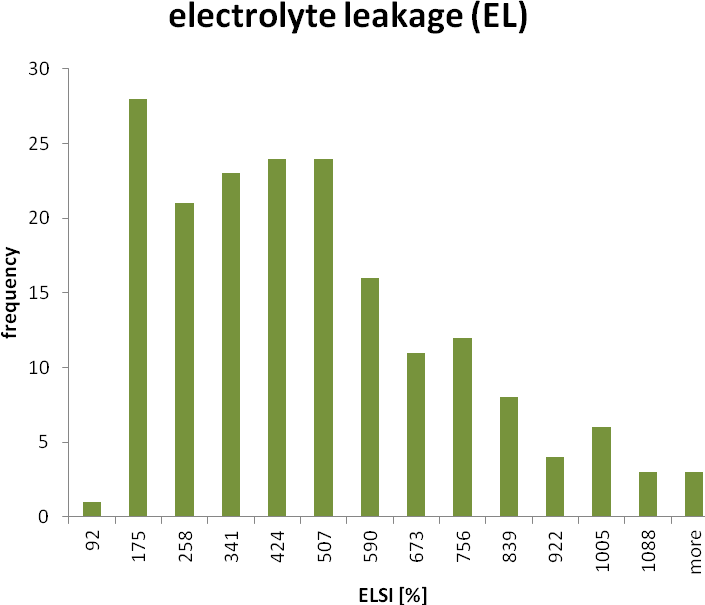


Fig S12. Histogram of stress index values for net photosynthetic rate (NPSI) measured during phenotyping of F_3_ progeny in malt-type barley mapping population.


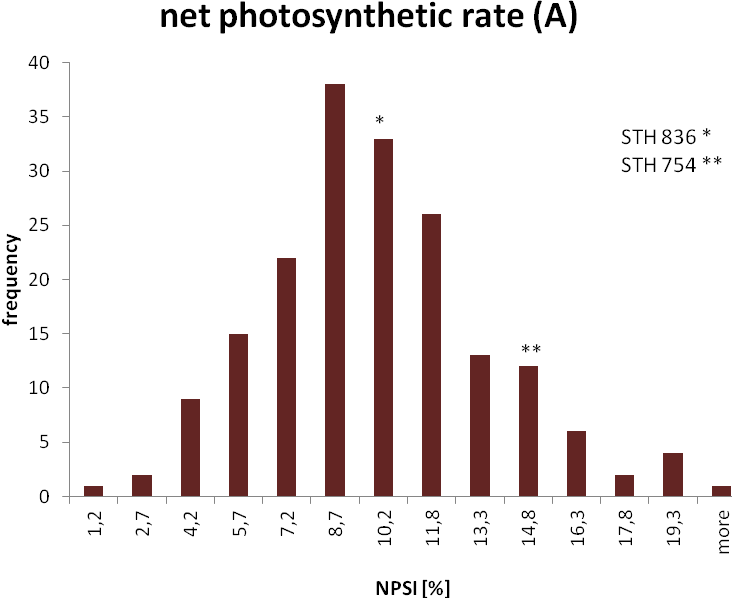


Fig S13. Histogram of stress index values for water content (WCSI) measured during phenotyping of F_3_ progeny in malt-type barley mapping population.

STH 836 *

STH 754 **

*


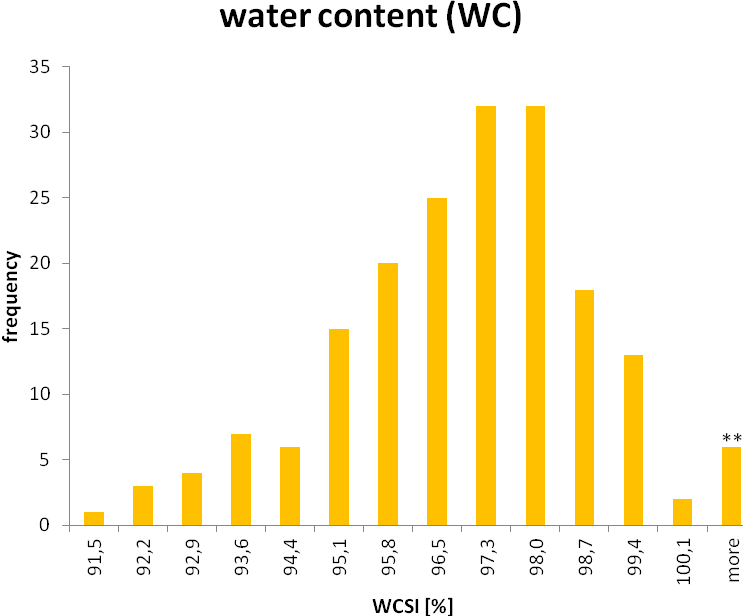


Fig S14. Histogram of stress index values for Fv'/Fm' (FSI) measured during phenotyping of F_3_ progeny in malt-type barley mapping population.

**

*

STH 836 *

STH 754 **


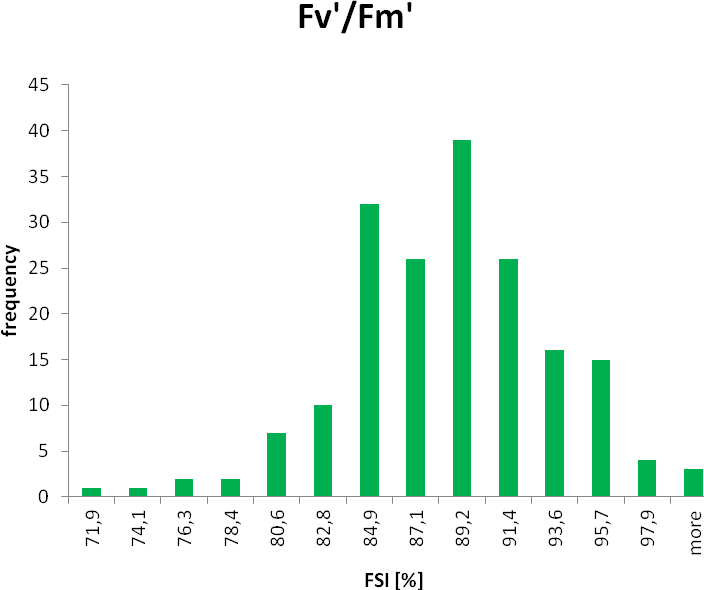


Fig S15. Histogram of stress index values for q_p_ (QSI) measured during phenotyping of F_3_ progeny in malt-type barley mapping population.

**

*


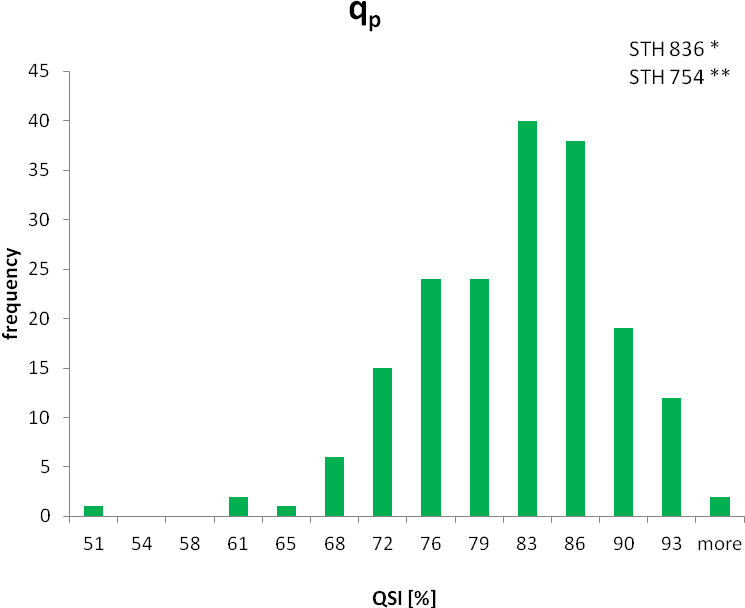


Fig S16. Histogram of stress index values for ΦPSII (PSSI) measured during phenotyping of F_3_ progeny in malt-type barley mapping population.

STH 836 *

STH 754 **


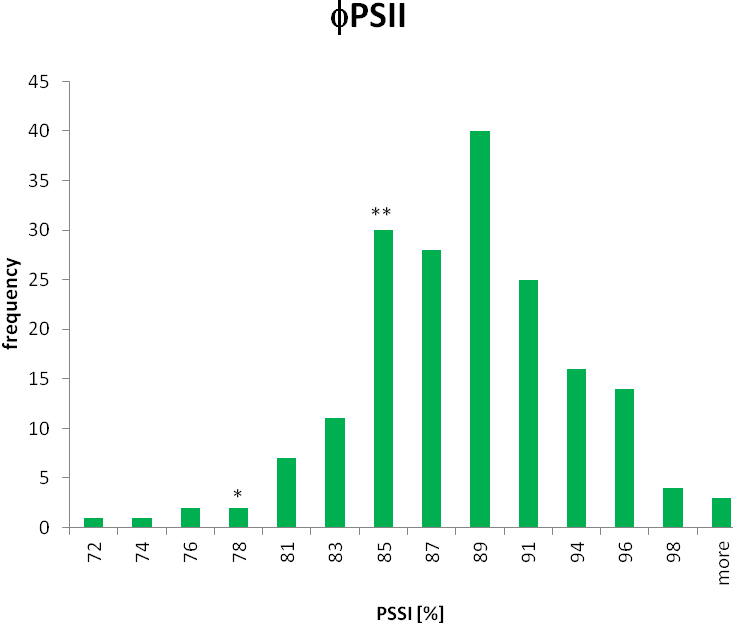


Fig S17. Histogram of stress index values for RC/CSm (RSI) measured during phenotyping of F_3_ progeny in malt-type barley mapping population.

STH 836 *

STH 754 **


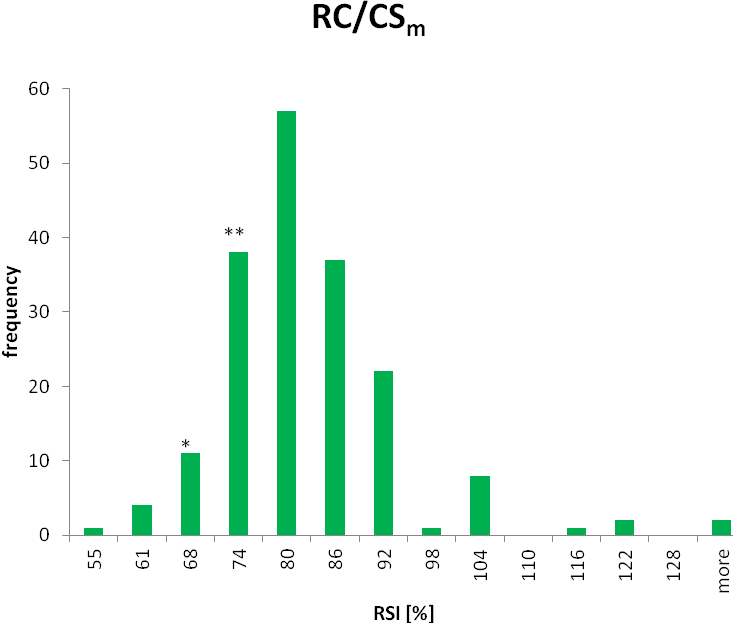


Fig S18. Histogram of stress index values for ABS/CSm (ASI) measured during phenotyping of F_3_ progeny in malt-type barley mapping population.

*

**


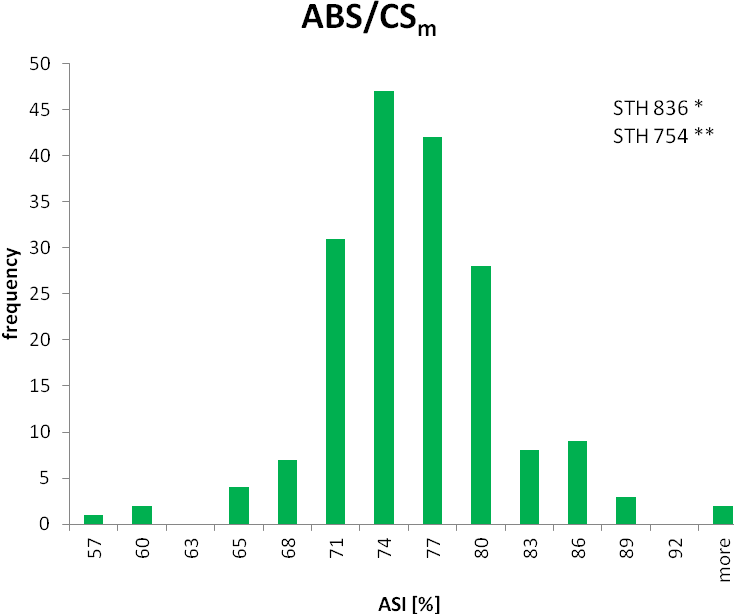


Fig S19. Histogram of stress index values for Tr_o_/CSm (TSI) measured during phenotyping of F_3_ progeny in malt-type barley mapping population.


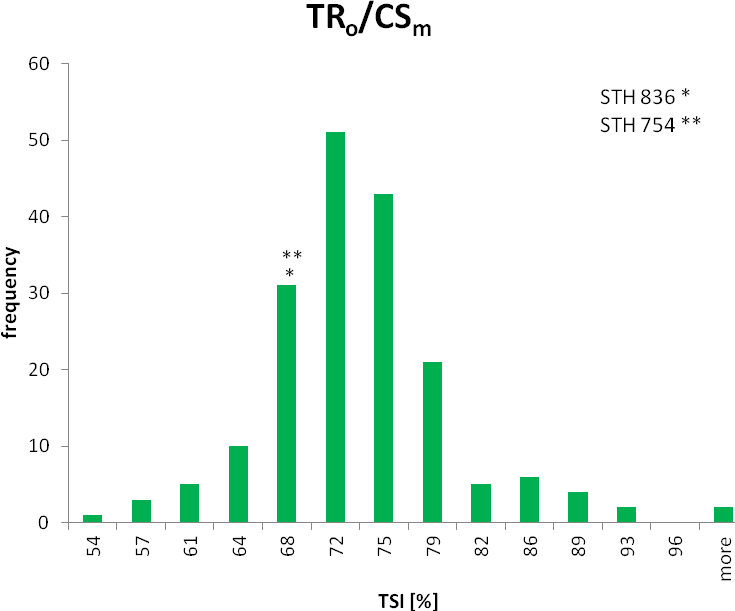


Fig S20. Histogram of stress index values for ET_o_ /CSm (ESI) measured during phenotyping of F_3_ progeny in malt-type barley mapping population.

STH 836 *

STH 754 **


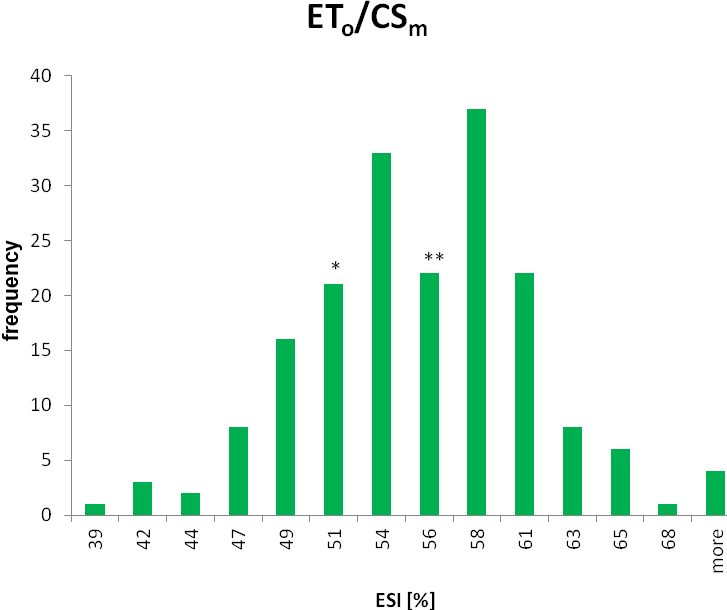


Fig S21. Histogram of stress index values for DI_o_/CSm (DSI) measured during phenotyping of F_3_ progeny in malt-type barley mapping population.

STH 836 *

STH 754 **


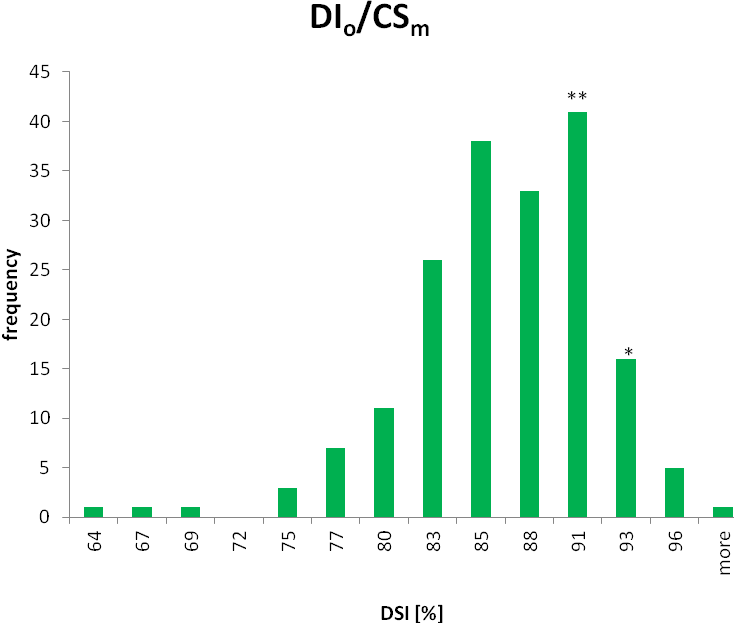


Fig S22. Histogram of stress index values for PI/CSm (PSI) measured during phenotyping of F_3_ progeny in malt-type barley mapping population.


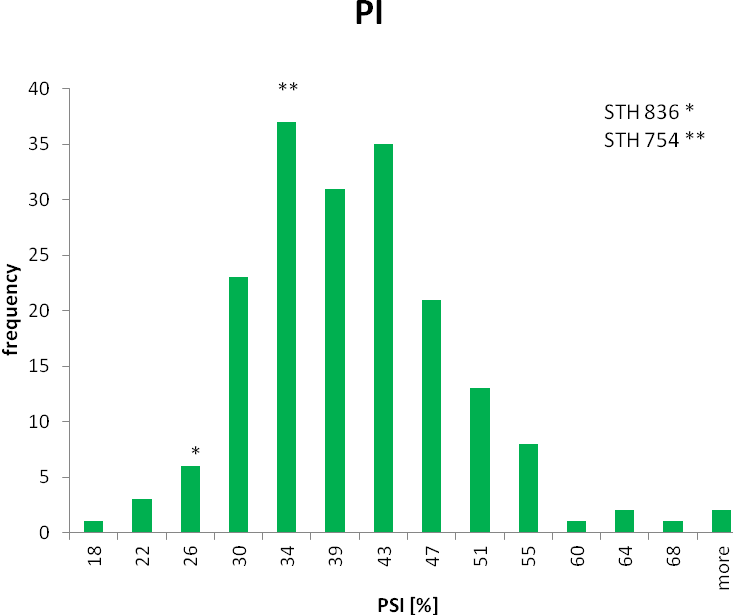


Fig S23. Histogram of stress index values for ΦPSII/ΦCO_2_ (CSI) measured during phenotyping of F_3_ progeny in malt-type barley mapping population.


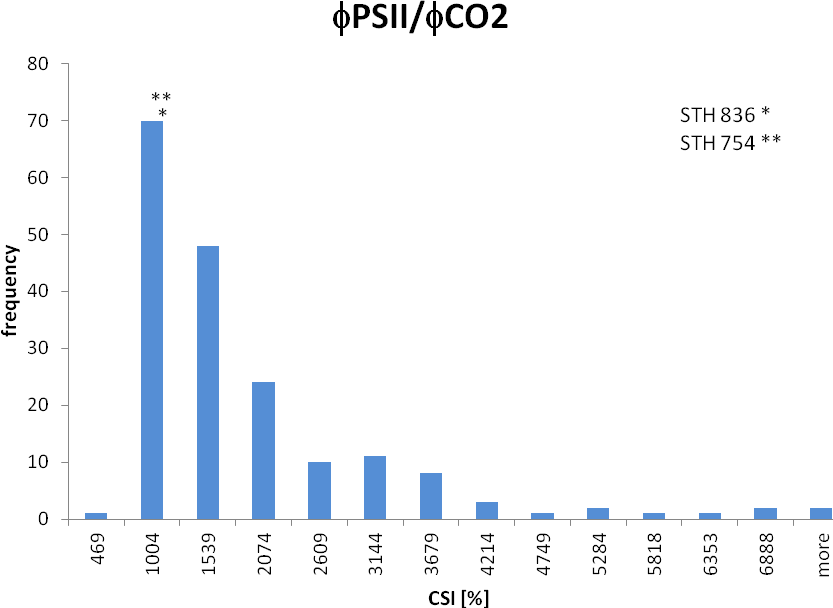


Fig S24. Histogram of stress index values for water content (WCSI) measured during phenotyping of F_3_ progeny in fodder-type barley mapping population.

MOB12055 *

STH369 **


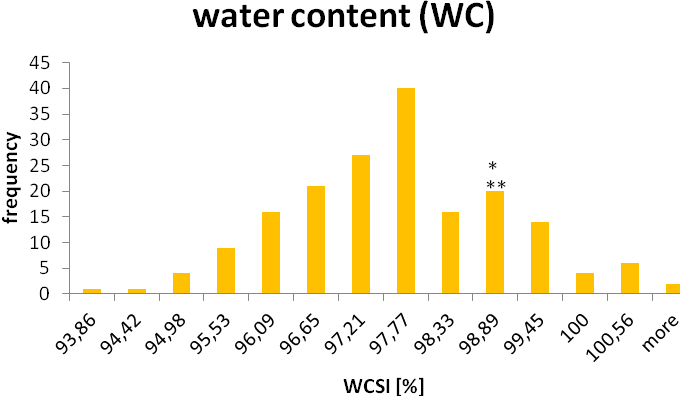


Fig S25. Histogram of stress index values for electrolyte leakage (ELSI) measured during phenotyping of F_3_ progeny in fodder-type barley mapping population.


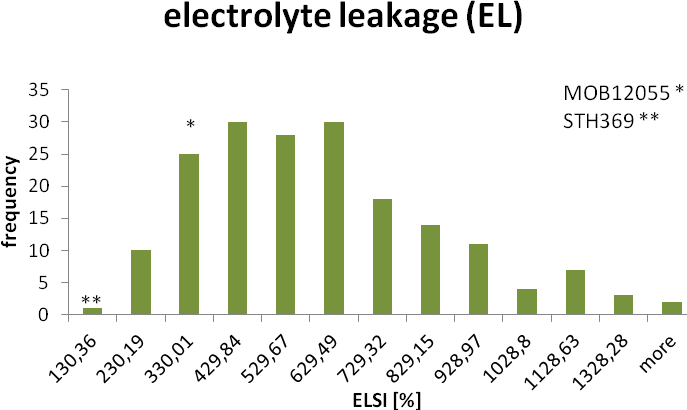


Fig S26. Histogram of stress index values for net photosynthetic rate (NPSI) measured during phenotyping of F_3_ progeny in fodder-type barley mapping population.


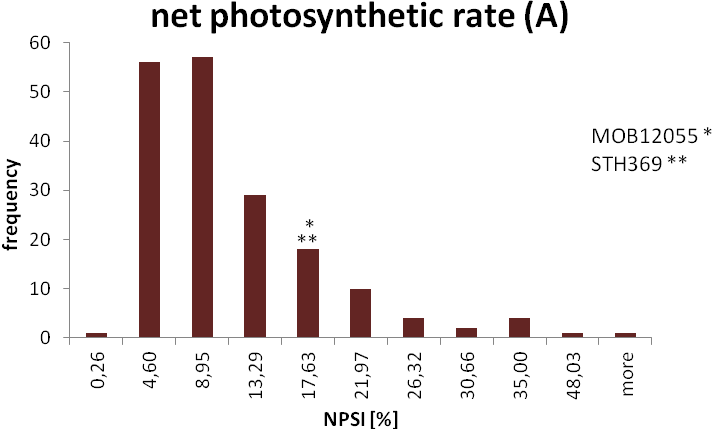


Fig S27. Histogram of stress index values for Fv'/Fm' (FSI) measured during phenotyping of F_3_ progeny in fodder-type barley mapping population.


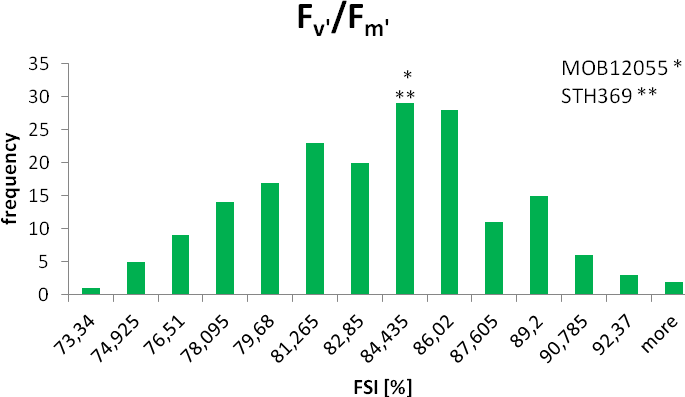


Fig S28. Histogram of stress index values for q_p_ (QSI) measured during phenotyping of F_3_ progeny in fodder-type barley mapping population.


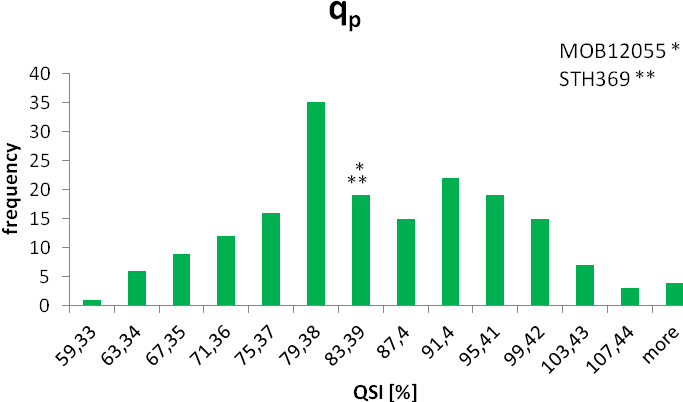


Fig S29. Histogram of stress index values for ΦPSII (PSSI) measured during phenotyping of F_3_ progeny in fodder-type barley mapping population.


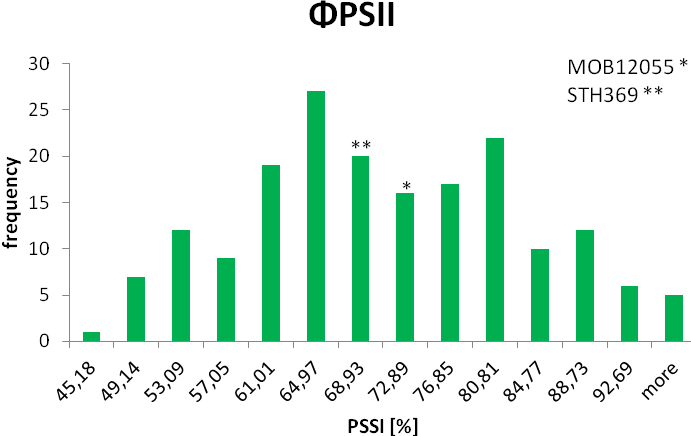


Fig S30. Histogram of stress index values for ΦPSII/ΦCO_2_ (CSI) measured during phenotyping of F_3_ progeny in fodder-type barley mapping population.


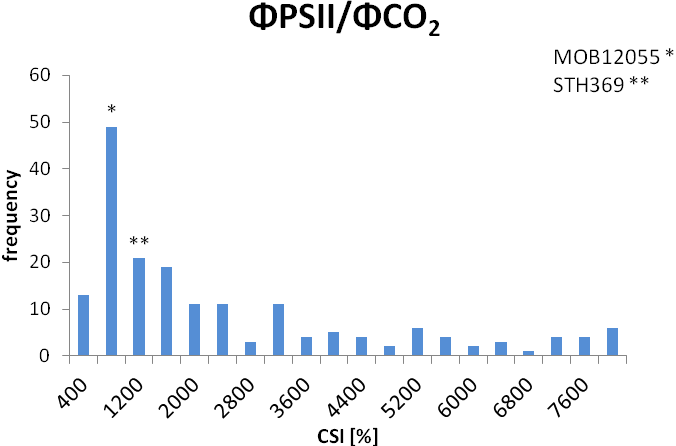


Table S1. SSR markers used for the saturation of genetic maps of malting and fodder barley.

| ***marker signature*** | ***primer sequence (5’-3’)*** | | ***Reference^*^*** |
| --- | --- | --- | --- |
|  | ***forward*** | ***reverse*** |  |
| Bmag0876 | AATTAAAAGCTGAAGGTCTACA | CTGCTCCTTCAACGACTAC | http://wheat.pw.usda.gov/cgi-bin/graingenes |
| EBmac405 | ATGTAGCTCGGAATGTGTAGT | CATGTTGGATAAGAGTAGAGGA | Varshney RK et al. (2007) |
| Bmag0872 | ATGTACCATTACGCATCCA | GAAATGTAGAGATGGCACTTG | Ramsay L et al. (2000) |
| Bmag211 | ACATCATGTCGATCAAAGC | ATTCATCGATCTTGTATTAGTCC | Sato K et al. (2009) |
| Bmag692 | GCAAGGTATCTCTTGTATTTTG | TGGCATCTACAATCTAAAACA | Ramsay L et al. (2000) |
| Bmag131 | CCTCCACACAAAAAATCC | TTTCAGAAACGGAGTTTTG | Ramsay L et al. (2000) |
| HVM44 | AAATCTCAGGTTCGTGGGCA | CCACGGAGACCACCTCACTT | Liou ZW et al. (1996) |
| Bmag136 | GTACGCTTTCAAACCTGG | GTAGGAGGAAGAATAAGGAGG | Ramsay L et al. (2000) |
| Bmac209 | ATGCCTGTGTGTGGACCAT | CTAGCAACTTCCCAACCGAC | Ramsay L et al. (2000) |
| Bmag6 | TGCAGTTACTATCGCTGATTTAGC | TTAAACCCCCCCCCTCTAG | Ramsay L et al. (2000) |
| Bmag603 | ATACCATGATACATCACATCG | GGGGGTATGTACGACTAACTA | Ramsay L et al. (2000) |
| Bmag500 | AATGTAAGGGAGTGTCCATAG | GGGAACTTGCTAATGAAGAG | Ramsay L et al. (2000) |
| Bmag206 | TAGAACTGGGTATTTCCTTGA | TTTTCCCCTATTATAGTGACG | Ramsay L et al. (2000) |
| EBmag794 | CAGTCATAACCTGATGAACAA | TCACACTTATCTTGCTGCTAA | Ramsay L et al. (2000) |
| Bmag7 | TCCCCTATTATAGTGACGGTGTG | TGAAGGAAGAATAAACAACCAACA | Ramsay L et al. (2000) |
| EBmac0713 | GGTAAAACATTTCCCTCGT | TAGAGATCACTCTCTTCTGTGC | Ramsay L et al. (2000) |
| HVM04 | AGAGCAACTACCAGTCCAATGGCA | GTCGAAGGAGAAGCGGCCCTGGTA | Saghai Maroof MA et al. (1994) |
| GBM1208 | CTACCGAGCTCCTCCTCCTC | GGCCTCCTTCTTGTCGTAGA | [http://wheat.pw.usda.gov/cgi-bin/graingenes](http://wheat.pw.usda.gov/cgi-bin/graingenes/report.cgi?class=probe&name=GBM1208) |
| Bmac0144 | ACTTATTCTGCATCCTGGGT | TACGTGTACATACTCTACGATTTG | http://wheat.pw.usda.gov/cgi-bin/graingenes |
| scssr02503 | AACAACTTTTGATGGACAAACC | TGTCTTTTCTTTTTGCTCTGC | http://wheat.pw.usda.gov/cgi-bin/graingenes |
| scssr02093 | CGTCACGCACACATCGAC | GATCTCTCCTCGGGCATC | http://wheat.pw.usda.gov/cgi-bin/graingenes |
| Bmac0047b | AACACACGTACACAAATACACA | ACGTCCATCACTTTGACC | [http://wheat.pw.usda.gov/cgi-bin/graingenes](http://wheat.pw.usda.gov/cgi-bin/graingenes/report.cgi?class=probe&name=GBM1208) |
| Bmag0867 | CCCCACACTGACCTACAG | TTACATCTGCTAGATCGAAGC | Hayden MJ et al. (2008) |
| GBM1400 | AGCGCTCTCTCACACACAGA | ATTCCTGCCTGTTTTTCGTG | [http://wheat.pw.usda.gov/cgi-bin/graingenes](http://wheat.pw.usda.gov/cgi-bin/graingenes/report.cgi?class=probe&name=GBM1208) |
| Bmag0210 | ACCTACAGTTCAATAGCTAGTACC | GCACAAAACGATTACATCATA | [http://wheat.pw.usda.gov/cgi-bin/graingenes](http://wheat.pw.usda.gov/cgi-bin/graingenes/report.cgi?class=probe&name=GBM1208) |
| Bmag0003 | GATCAAAGAGAACATGCGAT | GTAGTTCAGCATAGACCTACAGG | [http://wheat.pw.usda.gov/cgi-bin/graingenes](http://wheat.pw.usda.gov/cgi-bin/graingenes/report.cgi?class=probe&name=GBM1208) |
| Bmag0378 | ATCCAACTATAGTAGCAAAGCC | CTTTTGTTTCCGTAGCATCTA | [http://wheat.pw.usda.gov/cgi-bin/graingenes](http://wheat.pw.usda.gov/cgi-bin/graingenes/report.cgi?class=probe&name=GBM1208) |
| HvLOX | CACCCTTATTTATTGCCTTAA | CAGCATATCCATCTGATCTG | [http://wheat.pw.usda.gov/cgi-bin/graingenes](http://wheat.pw.usda.gov/cgi-bin/graingenes/report.cgi?class=probe&name=GBM1208) |
| Bmag0613 | AAGAACACCATATGATCCAAC | CTCCATGACTATGAGGAGAAG | [http://wheat.pw.usda.gov/cgi-bin/graingenes](http://wheat.pw.usda.gov/cgi-bin/graingenes/report.cgi?class=probe&name=GBM1208) |
| GBMS0180 | GGAACTAATGCTTCGGTCCA | TGGTGCAAGTGAGCACCTAC | [http://wheat.pw.usda.gov/cgi-bin/graingenes](http://wheat.pw.usda.gov/cgi-bin/graingenes/report.cgi?class=probe&name=GBM1208) |
| EBmac0602 | CCGTCTAGGGAGAGGTTCTC | GATTGGAGCTTCGGATCAC | [http://wheat.pw.usda.gov/cgi-bin/graingenes](http://wheat.pw.usda.gov/cgi-bin/graingenes/report.cgi?class=probe&name=GBM1208) |

^*^ Hayden MJ, Nguyen TM, Waterman A, Chalmers KJ (2008) Multiplex-Ready PCR: A new method for multiplexed SSR and SNP genotyping. BMC Genomics 9: 80

Liou ZW, Biyashev RM, Saghai Maroof MA (1996) Development of simple sequence repeat DNA markers and their integration into a barley linkage map. Theor Appl Genet 93: 869-876

Ramsay L, Macaulay M, degli Ivanissevich S et al. (2000) A simple sequence repeat-based linkage map of barley. Genetics 156: 1997-2005

Saghai Maroof MA, Biyashev,RM, Yang GP, Zhang Q, Allard RW (1994) Extraordinarily polymorphic microsatellite DNA in barley: species diversity, chromosomal locations, and population dynamics. Proc Natl Acad Sci USA 91: 5466-5470

Sato K, Nankaku N, Takeda K (2009) A high-density transcript linkage map of barley derived from a single population. Heredity 103: 110-117

Varshney RK, Marcel TC, Ramsay L et al. (2007) A high density barley microsatellite consensus map with 775 SSR loci. Theor Appl Genet 114: 1091
